# Effects of TiO_2_ nanoparticles on the kinetics and conformation of bovine alkaline phosphatase

**DOI:** 10.1016/j.bbrep.2026.102709

**Published:** 2026-07-18

**Authors:** Nasim Babaknejad, Behzad Shareghi, Ali Akbar Saboury

**Affiliations:** a Department of Biology, Faculty of Science, University of Shahrekord, Shahrekord, Iran; b Institute of Biochemistry and Biophysics, University of Tehran, Tehran, Iran

**Keywords:** Titanium dioxide nanoparticles, Bovine alkaline phosphatase, Spectroscopic analysis, Static quenching, Protein conformational change

## Abstract

This study observed the interaction between titanium dioxide (TiO_2_) nanoparticles and bovine intestinal alkaline phosphatase (BALP) through a combination of spectroscopic techniques and enzymatic activity assays. Fluorescence spectroscopy suggested that a static quenching mechanism governs the interaction, supported by the binding constant and site number determined at three temperatures. The predominant forces involved in the nanoparticle–protein complex were identified as hydrogen bonding and van der Waals interactions. Circular dichroism analysis indicated substantial alterations in both secondary and tertiary structures of BALP in the presence of TiO_2_. Moreover, enzymatic assays revealed that TiO_2_ acts as an uncompetitive inhibitor, reducing the enzyme's activity. These findings provide valuable insight into the conformational and functional consequences of TiO_2_ nanoparticle interaction with proteins.

## Highlights

• Titanium dioxide nanoparticle binds to the bovine alkaline phosphatase.
• Titanium dioxide nanoparticle quenches the bovine alkaline phosphatase fluorescence intensity by static binding mechanism.
• Titanium dioxide nanoparticle inhibits enzyme activity.
• Titanium dioxide nanoparticle changes enzyme secondary and tertiary structures of bovine alkaline phosphatase.

## Graphical abstract

**Figure:**
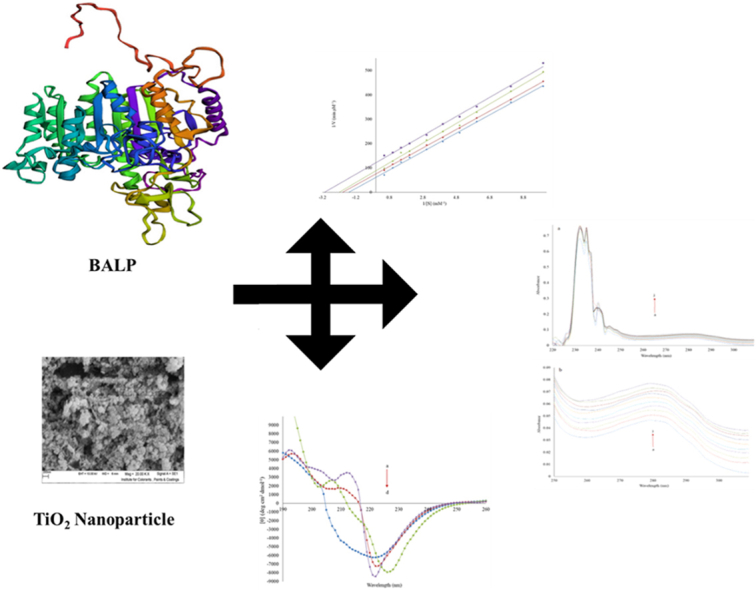

## 1 Introduction

Proteins serve as fundamental biomolecules with diverse roles, ranging from providing structural and facilitating mechanical functions to mediating cell signaling and catalyzing biochemical reactions [1]. Enzymes, as specialized proteins, play a vital role in regulating essential cellular processes, including metabolism, immune responses, gene expression, and signal transduction. Their utility spans numerous industries, particularly pharmaceuticals, food processing, environmental monitoring, and biomedical research [2,3].

Alkaline phosphatases (ALPs; EC 3.1.3.1) represent a family of metalloenzymes that hydrolyze phosphomonoesters to release phosphate and alcohol. Structurally, they are homodimeric and contain two zinc ions and one magnesium ion at active site, which are indispensable for catalytic activity. ALPs are evolutionarily conserved and are found across a wide spectrum of organisms, from bacteria to mammals [[4], [5], [6], [7]]. Mammalian ALPs typically exhibit higher catalytic efficiency, elevated Km values, alkaline pH optima, and reduced thermal stability compared to their bacterial counterparts. Clinically, ALP activity serves as a diagnostic biomarker for various pathological conditions, especially those involving liver and bone disorders. In the dairy industry, its activity is used to verify successful pasteurization processes [[8], [9], [10]]. Among mammalian ALPs, bovine intestinal alkaline phosphatase (BALP) is widely used in laboratory diagnostics such as enzyme-linked assays, nucleic acid detection methods, and polymerase chain reaction (PCR), due to its high specific activity [11,12].

Nanoparticles (NPs) have emerged as valuable tools in areas such as targeted drug delivery, biosensing, catalysis, imaging, and cancer therapy. The increasing prevalence of engineered nanoparticles has raised significant interest in understanding their interactions with biological macromolecules [[13], [14], [15], [16], [17], [18]]. Protein–nanoparticle interactions are governed by several physicochemical forces, including hydrogen bonding, van der Waals forces, and solvation effects, which depend largely on environmental pH and surface charge properties [[17], [18], [19]]. Despite extensive research, the detailed mechanisms underlying such interactions remain insufficiently understood and require further investigation [20].

TiO_2_ nanoparticles are among the most widely utilized nanomaterials due to their unique UV resistance, antibacterial properties, and photocatalytic activity. Their incorporation into consumer and industrial products such as cosmetics, paints, and environmental remediation systems has increased concerns regarding their biological impacts. Previous studies indicate that TiO_2_ nanoparticles can interact with proteins and enzymes, potentially altering their structure and function [21,22]. Therefore, comprehensive characterization of TiO_2_−protein interactions is crucial for advancing knowledge in nanotoxicology and nanomedicine [[23], [24], [25]].

This study explores the interaction TiO_2_ nanoparticles and BALP through a combination of spectroscopic techniques and enzymatic activity assays.

## 2 Results and discussion

### 2.1 UV–vis absorption spectra

**Fig. 1:**
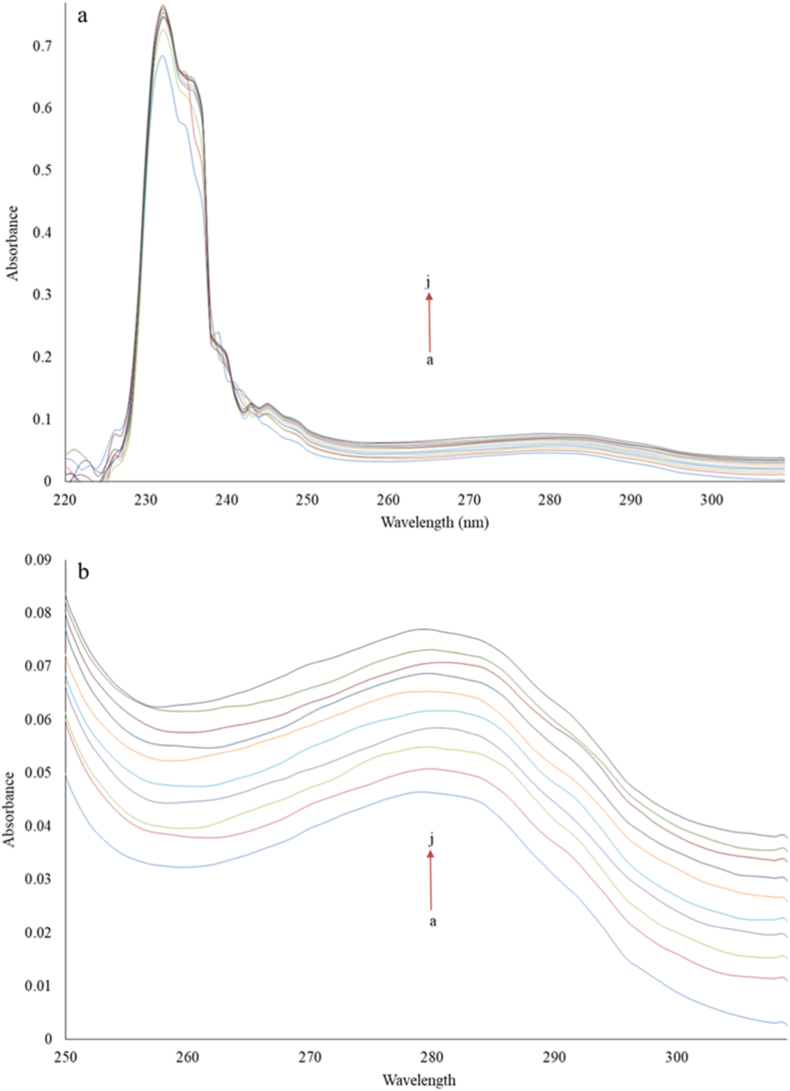
Bovine alkaline phosphatase spectra before and after interacting with titanium dioxide nanoparticles at 298 K. a: bovine alkaline phosphatase spectrum (200-400 nm), a→j: the difference spectrum of bovine alkaline phosphatase in the presence of different concentrations of titanium dioxide nanoparticles. C _bovine alkaline phosphatase_ = 6.06 × 10^−7^ M, a → j C _titanium dioxide_ = (0, 0.5, 1, 3, 5, 7, 9, 11, 13, 15 × 10^−6^ M). Thick blue line (a): BALP only (6.06 × 10^−7^ M, no TiO_2_).

The interaction between BALP and TiO_2_ nanoparticles was initially assessed through UV–visible spectroscopy to monitor potential conformational alterations in the enzyme. As depicted in Fig. 1, native BALP exhibited two characteristic absorption peaks in the near-UV region, primarily corresponding to the aromatic residues tryptophan and tyrosine, typically absorbing between 275 and 280 nm [[26], [27], [28]]. A strong absorption band observed between 210 and 240 nm reflects the peptide backbone's (C=O) contribution to the spectrum. Upon incremental addition of TiO_2_ nanoparticles, an increase in the absorbance at both 232 nm and 280 nm was observed. This enhancement suggests that TiO_2_ binding leads to an increased hydrophobic environment surrounding aromatic residues, indicative of structural rearrangements. Additionally, the spectral shifts at 232 nm imply modifications in the enzyme's secondary structure [29]. Overall, these changes reflect a direct interaction between TiO_2_ and BALP, likely resulting in conformational perturbations of the enzyme.

### 2.2 Fluorescence spectroscopy measurements

**Fig. 2:**
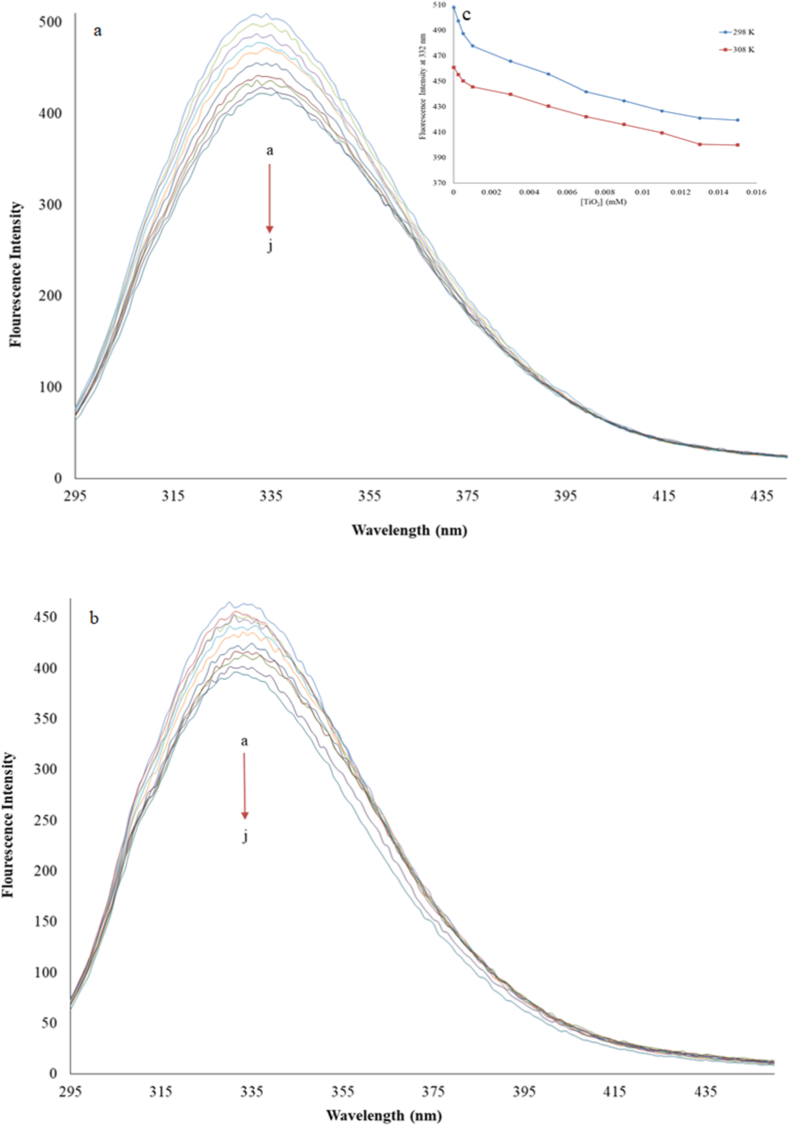
Fluorescence spectrum of bovine alkaline phosphatase before and after interaction with titanium dioxide nanoparticles at 298 K (a) and 308 K (b) (λex = 290 nm, and λem = 295-450). Insets (c): decrease of maximum fluorescence signal in the presence of the nanoparticle. C _bovine alkaline phosphatase_: 6.06 × 10^−7^ M, a → j C _titanium dioxide_ = (0, 0.5, 1, 3, 5, 7, 9, 11, 13, 15 × 10^−6^ M). Thick blue line (a): BALP only (6.06 × 10^−7^ M, no TiO_2_).

To further investigate the interaction between TiO_2_ nanoparticles and BALP, intrinsic fluorescence emission spectra were recorded at three temperatures (298 K, 308 K, and 318 K). Excitation at 290 nm, specific to tryptophan residues, resulted in an emission peak at 332 nm for the native enzyme, attributable to the presence of four tryptophan residues within the BALP structure [30,31]. Gradual addition of TiO_2_ led to a pronounced decrease in fluorescence intensity, along with a red shift of approximately 3 nm (from 332 to 335 nm), indicating alterations in the local environment of tryptophan residues. These observations imply that TiO_2_ nanoparticles interact with the enzyme and disrupt the hydrophobic core, causing conformational changes Fig. 2.

Analysis of the fluorescence quenching followed the Stern–Volmer model. Eq. (3) [31,32]:

(3) $$\frac{F_{0}}{F}=1+k_{q}\tau_{0}\left[Q\right]=1+K_{SV}\left[Q\right]$$

**Fig. 3:**
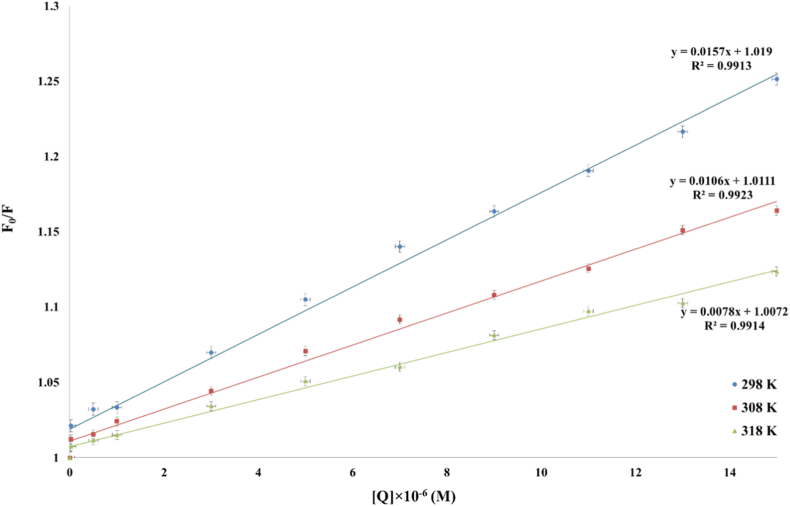
Stern–Volmer plots of bovine alkaline phosphatase in the presence of titanium dioxide at 298 K (●), 308 K (■), and 318 K (▲) (λex = 290 nm, λem = 295-450 nm). C _bovine alkaline phosphatase_: 6.06 × 10^−7^ M, C _titanium dioxide_ = (0, 0.5, 1, 3, 5, 7, 9, 11, 13, 15 × 10^−6^ M). The y-axis starts at 1 because F_0_/F = 1 at [Q] = 0 according to the Stern–Volmer relation.

In this equation, *F*_*0*_ and *F* are fluorescence signals in the absence and presence of quencher (TiO_2_ nanoparticles), respectively; *K*_*SV*_ is the Stern–Volmer quenching constant, *Q* is the molar concentration of quencher, *k*_*q*_ is the quenching rate constant, and *τ*_*0*_ is the average lifetime of BALP without the quencher. *τ*_*0*_ for bio-macromolecules is 10^−8^ s [33]. The *K*_*SV*_ was determined by determining the slope of a linear regression of plot *F*_*0*_*/F* vs. [*Q*] at three temperatures (Fig. 3). The equation $k_{q}=K_{SV}/\tau_{0}$ was used to calculate the quenching rate constant, *k*_*q*_ [31]. Table 1 summarizes Stern–Volmer quenching constants of BALP-TiO_2_ complex at 298 K and 308 K at pH 9.5. A linear relationship was obtained between F_0_/F and quencher concentration, confirming the quenching mechanism. Notably, the quenching constants (*K*_*SV*_) decreased with increasing temperature, consistent with a static quenching process [34]. Furthermore, the calculated bimolecular quenching constants (*kq*) significantly exceeded the diffusion-limited maximum (∼2 × 10^10^ M^−1^s^−1^), further supporting the formation of a stable ground-state complex between BALP and TiO_2_ nanoparticles [31]. These results corroborate the UV–vis findings and underscore a static binding interaction, suggesting that TiO_2_ induces structural rearrangements that are thermodynamically stable at lower temperatures [15].

**Table 1:** Stern–Volmer quenching constants of bovine alkaline phosphatase-titanium dioxide complex.

*T* (K)	*K*_sv_ ×10^4^ (M^−1^)	*k*_q_ ×10^12^ (M^−1^s^−1^)	R^2^
298	1.57 ± 0.04	1.57 ± 0.04	0.9913
308	1.06 ± 0.03	1.06 ± 0.03	0.9923
318	0.7 ± 0.02	0.7 ± 0.02	0.9914

T: Absolute temperature; K_sv_: quenching constant; K_q_: quenching rate constants; R: correlation coefficient for K_sv_ values. Averages of triplicate determinations with SD values are shown.

### 2.3 Binding parameters of TiO_2_ nanoparticles to BALP

To determine the binding strength and stoichiometry of the BALP–TiO_2_ interaction, the double-logarithmic equation was employed, Eq. (4) [32]:

(4) $$\log\left(\frac{F_{0}-F}{F}\right)=\log\;K_{b}+n\;\log\left[Q\right]$$

**Fig. 4:**
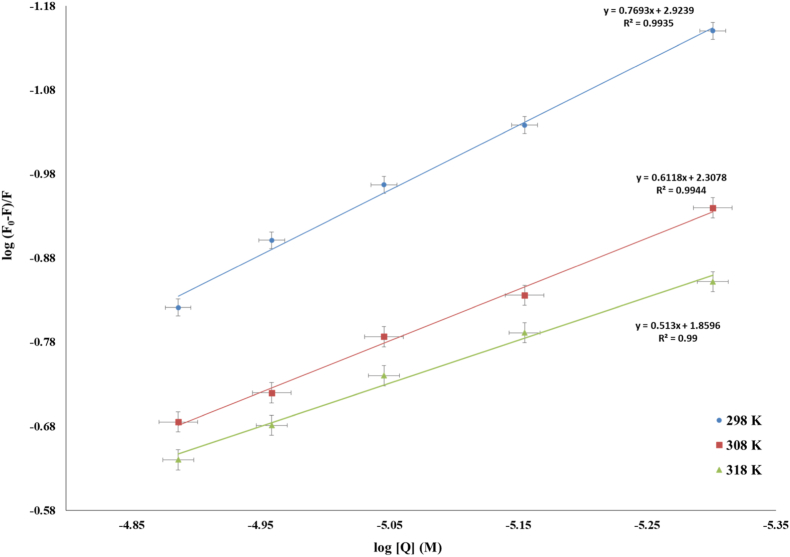
Plot of log [(*F*_*0*_*– F)/F*] *vs.* log [*Q*] of bovine alkaline phosphatase in the presence of titanium dioxide at 298 K (●) and 308 K (■), and 318 K (▲) (λex = 290 nm, λem = 295-450 nm). C _bovine alkaline phosphatase_: 6.06 × 10^−7^ M, C _titanium dioxide_ = (0, 5, 7, 9, 11, 13 × 10^−6^ M).

The plot of log [(F_0_–F)/F] versus log [Q] at three temperatures (298 K, 308 K, and 318 K) allowed for the estimation of the binding constant (Kb) and the number of binding sites (n) (Fig. 4 and Table 2). The data revealed a decrease in Kb values with increasing temperature, indicating that the stability of the BALP–TiO_2_ complex diminishes under thermal stress.

**Table 2:** Thermodynamic parameters of bovine alkaline phosphatase-titanium dioxide nanoparticle complex.

T (K)	*K*_*b*_ ×10^3^ (M^−1^)	*n*	R^2^	ΔH° (kJ mol^−1^)	ΔG° (kJ mol^−1^)	ΔS^°^ (J mol^−1^ K^−1^)
298	8.39 ± 0.50	≈1	0.993	−95.58 ± 5.5	−16.75 ± 1.21	−265.20 ± 7.10
308	2.03 ± 0.01	≈1	0.994	−95.58 ± 4.6	−13.90 ± 1.12	−265.20 ± 8.11
318	0.75 ± 0.01	≈1	0.990	−95.58 ± 4.8	−11.25 ± 0.81	−265.20 ± 6.21

T: Absolute temperature; K_b_: association constant; n: number of binding sites; R: correlation coefficient for K_b_ values; ΔG: Gibbs free-energy change; ΔH: enthalpy change; ΔS: entropy change. Averages of triplicate determinations with SD values are shown.

Furthermore, the calculated binding site values were close to one at both temperatures, suggesting a single predominant binding site for TiO_2_ on the BALP surface. This is consistent with a specific, saturable interaction rather than nonspecific aggregation.

### 2.4 Thermodynamic properties

Thermodynamic parameters governing the binding process were calculated using the Van't Hoff equation and plot (Fig. S1).

(5) $$\ln\;K_{b}=-\frac{\Delta Hᵒ}{RT}+\frac{\Delta Sᵒ}{R}$$

Where R is the gas constant, and T represents the absolute temperature. The values of ΔH° and ΔS° were obtained from the slope and intercept of the plot, respectively. The Gibbs free energy change (ΔG°) at each temperature was subsequently calculated using the Gibbs–Helmholtz equation:

(6) ΔGᵒ=ΔHᵒ−TΔS

The negative ΔG° values observed at both temperatures verified that the BALP–TiO_2_ interaction is spontaneous. Additionally, the significantly negative enthalpy change (ΔH°) and the negative entropy change (ΔS°) suggest that the binding is driven by enthalpy and involves a decrease in system randomness, and is dominated by short-range interactions rather than hydrophobic or electrostatic forces [35]. The predominant intermolecular forces governing the BALP–TiO_2_ interaction can be deduced from the signs of the thermodynamic parameters. According to Ross and Subramanian (1981), such negative ΔH° and ΔS° values are characteristic of hydrogen bonding and van der Waals interactions, where complex formation leads to reduced system randomness [36]. A negative ΔH° accompanied by a positive ΔS° is characteristic of electrostatic interactions as the primary driving force. Finally, positive ΔH° and ΔS° values are classically associated with hydrophobic effects as the main stabilizing factor [36].

This interpretation is further supported by molecular docking analysis, which identified several hydrogen bonds (2.6–3.0 Å) and numerous van der Waals contacts between surface residues, such as Ser, Thr, and Tyr, and TiO_2_ clusters. Together, these thermodynamic and computational findings confirm that hydrogen bonding and van der Waals forces are the main contributors to the TiO_2_-BALP binding mechanism under alkaline conditions.

The isoelectric point (pI) of BALP is 5, indicating that at pH 9.5, it carries a negative net charge. The pI of TiO_2_ is approximately 6. Consequently, at a pH of 9.5 in the DEA buffer, it is also considered negative. Thus, this further supports the likelihood that van der Waals and hydrogen bonding interactions serve as the primary binding forces between the TiO_2_ nanoparticle and BALP [40].

### 2.5 Circular dichroism spectroscopy

#### 2.5.1 Far-UV CD

**Fig. 5:**
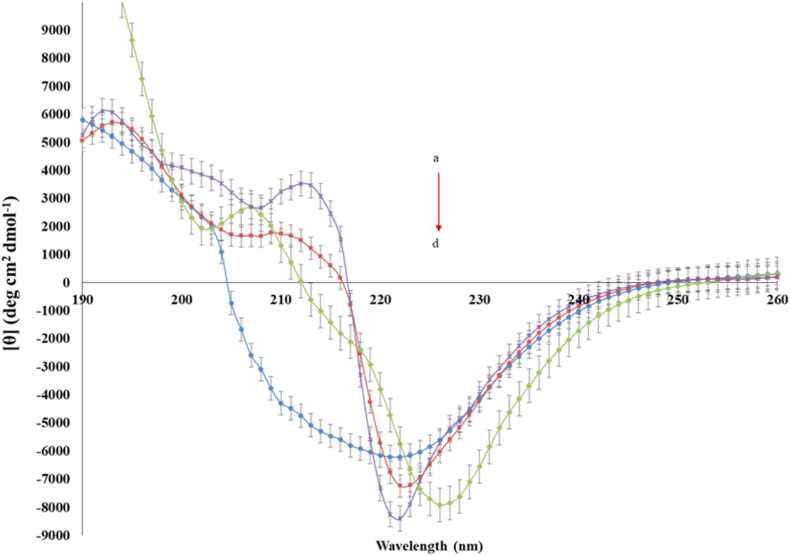
Far-ultraviolet circular dichroism spectra of bovine alkaline phosphatase-titanium dioxide nanoparticles (a → d C _titanium dioxide_: 0 (●), 1(■), 5(♦), 9 (×) ×10^−6^ Μ) (298 K). C _bovine alkaline phosphatase_: 6.06 × 10^−7^ M [θ]: mean-residual ellipticity cm^2^ dmol^−1^. Thick blue line (●): BALP only.

Fig. 5 represents the CD spectrum of free BALP and BALP in the presence of different concentrations of TiO_2_ nanoparticles. Far-UV CD spectra (190–240 nm) provided insights into alterations in the enzyme's secondary structure upon TiO_2_ exposure. Native BALP exhibited characteristic α-helical signatures with minima near 208 and 222 nm [] [41]. Upon addition of TiO_2_ nanoparticles, a significant reduction in ellipticity at 222 nm was observed [37,38]. The intense negative bands at 208 and 222 nm are characteristic of α-helical secondary structure, while the weaker positive band at ∼195 nm and the overall spectral shape further support a predominantly helical conformation.

Accordingly, secondary-structure deconvolution was performed using CDNN v2.1 (Aviv software) on triplicate spectra, and the results (mean ± SD) are given in Table 3. Table 3 demonstrated a consistent decline in α-helix percentage and a corresponding increase in β-sheet and random coil structures, signifying that TiO_2_ binding induces protein unfolding or rearrangement [39,40]. A decrease at 222 nm (in conjunction with the 208 nm signal) is diagnostic of loss of α-helical content rather than indicative of β-sheet formation. The analysis shows a clear reduction in α-helix percentage with a concomitant increase in random coil and β-sheet components, consistent with partial unfolding of BALP upon interaction with TiO_2[_41]. All spectra were converted to mean residue ellipticity ([θ], deg·cm^2^·dmol^−1^) and baseline-corrected using TiO_2_ + buffer blanks. The secondary-structure percentages obtained from CDNN deconvolution were normalized to ensure that the total content (α-helix + β-sheet + β-turn + random coil) equals 100% for each condition. Minor deviations in the initial CDNN output were due to rounding of fractional values, and normalization does not affect the observed structural trends [[42], [43], [44]].

**Table 3:** Far ultra-violet circular dichroism data and secondary structures of bovine alkaline phosphatase (in %) in the absence and presence of titanium dioxide nanoparticle concentrations.

TiO_2_ concentration (μM)	α-Helix (%)	Antiparallel β-sheet (%)	β-Sheet (%)	β-Turn (%)	Random coil (%)
0	19.4 ± 1.4	12.9 ± 0.8	23.9 ± 1.4	19.6 ± 1.4	44.2 ± 2.5
1	13.3 ± 0.8	17.6 ± 0.9	34.3 ± 2.1	21.7 ± 1.4	51.1 ± 2.6
5	13.0 ± 1.2	17.5 ± 0.9	35.3 ± 1.5	22.3 ± 1.6	51.9 ± 2.6
9	12.7 ± 0.9	18.4 ± 1.0	35.6 ± 1.8	22.1 ± 1.5	53.2 ± 2.0

Secondary structure contents of BALP in the presence of various concentrations of TiO_2_ nanoparticles, as determined by far-UV CD spectroscopy. Values are normalized to sum to 100%. Data represent mean ± SD (n = 3).

#### 2.5.2 Near-UV CD

**Fig. 6:**
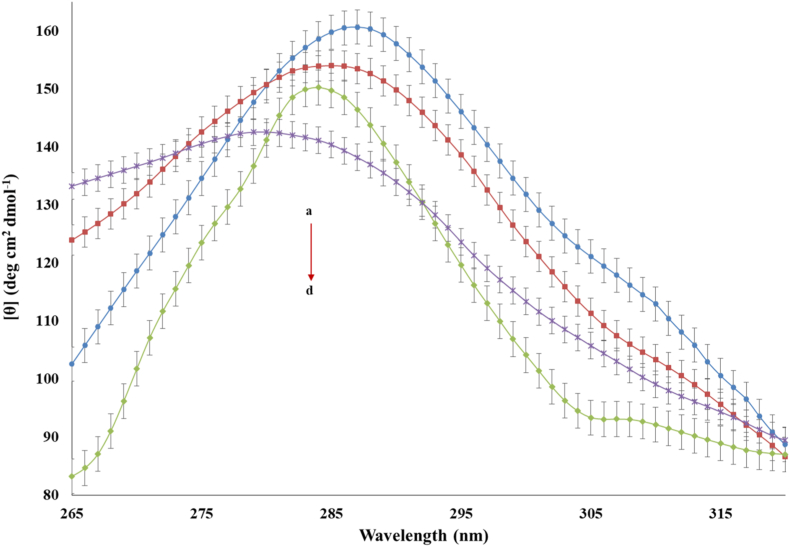
Near-ultraviolet circular dichroism spectra of bovine alkaline phosphatase-titanium dioxide nanoparticles (a → d C _titanium dioxide_: 0 (●), 3(■), 7(♦), 11 (×) ×10^−6^ Μ) (298 K). C _bovine alkaline phosphatase_: 6.06 × 10^−7^ M [θ]: mean-residual ellipticity cm^2^ dmol^−1^.

The near-UV band of proteins (250–320 nm) displays aromatic side-chain environment and interactions, and band intensity is affected by oligomerization and/or local conformational changes close to these residues. Consequently, this can be used to study changes of amino acid residues and the tertiary structure of proteins [42,43,45]. CD intensity of BALP in the presence of different concentrations of TiO_2_ nanoparticles was decreased in comparison with the free BALP (Fig. 6). Closer contact of chromophores with each other leads to the maintenance or increase of band intensity [42]. However, the intensity of these bands decreases by increasing the distance between aromatic acid residues increases. Then, TiO_2_ nanoparticles interacted with BALP and affected the tertiary structure of this enzyme. Nonetheless, due to the presence of aromatic acid residues and cysteine in the enzyme's structure, determining the precise reason for changes in the near-UV band is challenging. The observed discrepancy between the calculated binding energies and experimental data is problematic, indicating that further computational refinement or additional experimental validation may be required [[42], [43], [44]].

### 2.6 Effects of TiO_2_ nanoparticles on BALP kinetic parameters

**Fig. 7:**
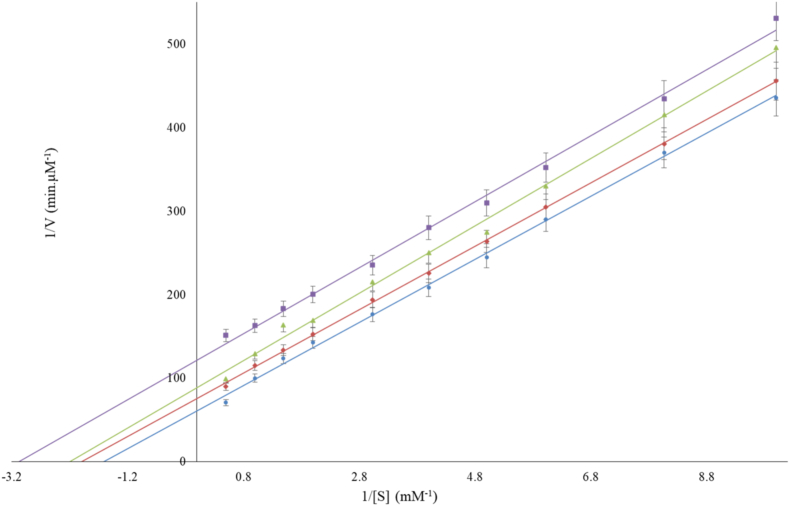
Lineweaver-Burk plot for the hydrolysis of p-nitrophenyl phosphate by bovine alkaline phosphatase at various concentrations of titanium dioxide nanoparticles at 298 K (pH = 9.5). C _titanium dioxide_ = 0 (●), 3(♦), 7(▲), 11 (■) ×10^−6^ M, C _bovine alkaline phosphatase_: 6.06 × 10^−9^ M, C _p-nitrophenyl phosphate_: 0, 124, 166, 200, 250, 330, 500, 670, 1000, 2000 × 10^−3^ M.

It was observed that the presence of TiO_2_ nanoparticles reduced the catalytic activity of BALP. As shown in Fig. 7 and Table 4, both Km and Vmax values decreased progressively with increasing concentrations of TiO_2_ nanoparticles. This pattern indicates that TiO_2_ nanoparticles interact with both the enzyme and the enzyme–substrate complex, suggesting an uncompetitive inhibition mechanism. Additionally, TiO_2_ nanoparticles lowered the kcat value while having no significant impact on the kcat/Km ratios (Table 4). The inhibitor constant (Ki) was determined from the secondary plots characteristic of uncompetitive inhibition and was calculated to be 9.52 μM (Fig. S2). Previous studies have similarly reported TiO_2_-mediated inhibition of lysozyme and trypsin [25,44]. These results are also in agreement with UV–visible, CD, and fluorescence spectroscopy analyses, which showed that nano-TiO_2_ interacts with BALP via van der Waals forces and hydrogen bonding. Such interactions lead to a reduction in α-helix content, reflecting partial unfolding of the enzyme's secondary structure. Collectively, these findings suggest that nano-TiO_2_ binding induces conformational changes in BALP, increasing the enzyme's affinity toward the nanoparticles rather than its substrate.

**Table 4:** The kinetic values of bovine alkaline phosphatase in the absence and presence of titanium dioxide nanoparticles.

Titanium dioxide nanoparticle concentration (μM)	*K*_m_×10^3^ (μM)	*k*_cat_ (min^−1^)	*V*_max_ (μM.min^−1^)	*k*_cat_/*K*_m_ (min^−1^.μM^−1^)
0	0.61 ± 0.02	2.62 ± 0.13	0.016 ± 0.001	4.24 ± 0.09
1	0.51 ± 0.21	2.15 ± 0.11	0.013 ± 0.000	4.21 ± 0.02
5	0.46 ± 0.10	1.94 ± 0.41	0.011 ± 0.000	4.21 ± 0.05
9	0.35 ± 0.00	1.46 ± 0.10	0.008 ± 0.000	4.19 ± 0.08

K_m_: Michaelis-Menten constant; k_cat_: turnover number; V_max_: maximum velocity; k_cat_/K_m_: catalytic efficiency of the enzyme.

### 2.7 Molecular docking studies

**Fig. 8:**
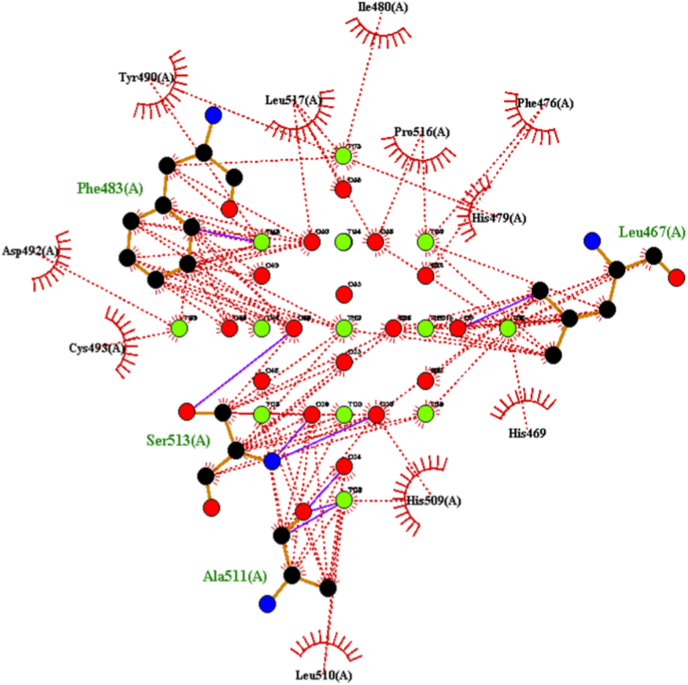
Graphical representation of BALP-TiO2 nanoparticle complex. Residues involved in hydrophobic interactions and Hydrogen Bonds are shown in graphical form.

**Fig. 9:**
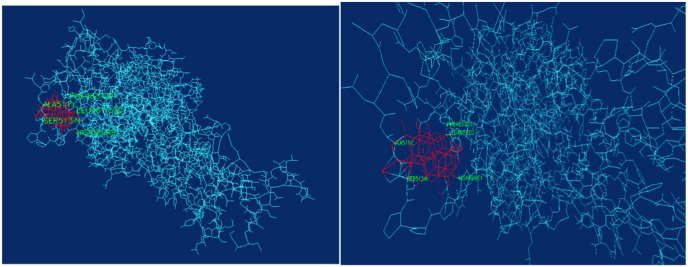
Interaction of TiO_2_ (red color) with BALP.

PatchDock was utilized for conducting molecular docking analyses. PatchDock (20) is a docking technique based on shape that employs principles of complementarity along with spherical harmonics to evaluate geometric feature-based shape alignment (15). A clustering RMSD of 4.0 Å was selected. The PatchDock score (PDS) and atomic contact energy (ACE) are presented in Table 5. It can be noted that the binding affinity of nano-TiO_2_ is 7654, with a minimal ACE value of −97.26 kJ mol^−1^. The area and transformation measurements were 482 and (2.72, 0.45, 2.59, 28.30, 25.08, and −5.86), respectively. Fig. 8 represents H-bond and hydrophobic analysis by using the Ligplot^+^ tool for nano-TiO_2_ and BALP, and Fig. 9 shows the interaction of nano-TiO_2_ with the BALP. Docking results suggested that hydrogen bonds and van der Waals forces were responsible for interactions between TiO_2_ nanoparticles and BALP.

**Table 5:** The main parameters derived from the PatchDock analysis.

Parameter	Description/Result
Score	7654
Scoring function (ACE)	−97.26 kJ mol^−1^
Area	956.20
Binding region	Active-site cleft near the catalytic Zn^2+^ center of BALP
Key interacting residues	Ser102, Thr104, Tyr127, Asn153, Asp327, His331, Lys340
Dominant interaction types	Hydrogen bonding and van der Waals interactions
Number of hydrogen bonds	5 (average, based on LigPlot + output)
Approximate H-bond distances	2.6–3.0 Å
Hydrophobic/van der Waals contacts	Observed around Tyr127, His331, and Lys340 residues
Docking pose stability	Stable, well-packed orientation in the catalytic pocket consistent with static quenching behavior
Interpretation	The predicted binding pattern supports the enthalpy-driven mechanism observed experimentally, confirming hydrogen bonding and van der Waals forces as major contributors.

To facilitate clearer interpretation, the main parameters derived from the PatchDock analysis are summarized in Table 5. The docking results revealed that TiO_2_ nanoparticles bind within the active-site cleft of BALP, forming multiple hydrogen bonds (2.6–3.0 Å) and van der Waals contacts with polar residues such as Ser102, Thr104, Tyr127, and Asp327. The calculated atomic contact energy (ACE = −97.26 kJ mol^−1^) indicates a favorable and stable complex formation. These findings are consistent with the experimental thermodynamic data, suggesting an enthalpy-driven binding mechanism dominated by hydrogen bonding and van der Waals interactions. It is well established that docking scoring functions significantly overestimate binding affinities and should not be directly compared with experimental ΔG° values.

## 3 Conclusion

This study comprehensively explored the interaction between titanium dioxide nanoparticles and bovine intestinal alkaline phosphatase using a multi-technique approach. Spectroscopic analyses demonstrated that TiO_2_ nanoparticles bind to BALP through a static quenching mechanism, leading to changes in the protein's microenvironment and secondary structure. Thermodynamic assessments confirmed a spontaneous, enthalpy-driven interaction dominated by hydrogen bonding and van der Waals forces. CD spectroscopy revealed significant alterations in both α-helical and tertiary structures, consistent with partial unfolding. Enzyme kinetics indicated that TiO_2_ acts as an uncompetitive inhibitor, reducing catalytic activity without entirely blocking substrate binding. Molecular docking results supported these findings, highlighting specific protein–nanoparticle interactions. Given the increasing use of TiO_2_ in various industrial and biomedical applications, understanding its influence on enzyme structure and function is critical for evaluating its biocompatibility and potential toxicity. This study offers valuable insights into the structural and functional consequences of protein–nanoparticle interactions.

## 4 Experimental

### 4.1 Materials

Bovine intestinal alkaline phosphatase, diethanolamine (DEA), magnesium chloride (MgCl_2_), and *p*-nitrophenyl phosphate (pNPP) were procured from Sigma-Aldrich (St. Louis, MO, USA). Titanium dioxide (TiO_2_) nanoparticles in the rutile form were purchased from US Research Nanomaterials Inc. (Houston, TX, USA). All reagents were of analytical grade and used without further purification.

### 4.2 Preparation of solutions

A 1 mg/mL BALP stock solution was prepared in 1.0 M DEA-HCl buffer (pH 9.5), supplemented with 0.5 mM MgCl_2_. TiO_2_ nanoparticles were dispersed in deionized water and sonicated in three 10-min cycles to ensure uniform suspension. The substrate pNPP was dissolved in the same DEA buffer at a final concentration of 4 mM and stored at 4°C.

### 4.3 Ultraviolet-visible absorption spectra

UV–vis spectra were recorded on an Ultrospec 4000 spectrophotometer (Pharmacia) using 1.0 cm quartz cuvettes. Absorbance readings were collected over the 200–500 nm range with a BALP concentration of 0.1 mg/mL in the absence and presence of varying concentrations of TiO_2_.

### 4.4 Fluorescence spectroscopy measurements

The fluorescence spectra were analyzed using a Shimadzu RF-5301 Fluorescence Spectrophotometer (Tokyo, Japan) that features a xenon lamp, a 1 cm quartz cell, and slit widths of 3 nm/5 nm. The BALP fluorescence emission spectra were recorded over a wavelength range from 300 to 450 nm. The excitation wavelength used was 290 nm. A circulating bath was employed to maintain a consistent temperature (298, 308, and 318 K) throughout the experiment. The inner filter effect (IFE) refers to the decrease in fluorescence intensity of a fluorophore due to the absorption of the excitation or emission light by the fluorophore itself or another material. To account for any inner filter effects in the fluorescence spectra, the following equation was applied, Eq. (1):

(1) $${\;F}_{cor}=F_{obs}\times e^{\frac{{OD}_{ex}+{OD}_{em}}{2}}$$

The corrected and observed fluorescence intensities are represented by F_cor_ and F_obs_, respectively, while OD_ex_ and OD_em_ denote the absorption of the system at the excitation and emission wavelengths, respectively. The fluorescence signals analyzed in this study are those that have been corrected.

### 4.5 Circular dichroism spectroscopy

CD measurements were conducted using an Aviv Model 215 spectropolarimeter. Far-UV spectra (190–260 nm) were recorded using 1 mm path length quartz cuvettes, while near-UV spectra (260–320 nm) employed 10 mm cuvettes. Enzyme concentration was maintained at 1 mg/mL. Secondary structure estimations were computed using the CDNN software (version 2.1), and data were reported as mean residue ellipticity [θ] in deg·cm^2^·dmol^−1^.

### 4.6 Enzymatic kinetic assay

Kinetic assays were performed at 308 K using a Shimadzu UV-2600 spectrophotometer. Reactions were initiated by adding 20 μL of enzyme solution to 2 mL of assay buffer containing varying concentrations of p-nitrophenyl phosphate (pNPP). Hydrolysis was monitored at 405 nm by measuring the formation of *p*-nitrophenol (*ε* = 18.5 × 10^3^ M^−1^ cm^1^). The Michaelis–Menten constants (K_m_ and V_max_) were derived from Lineweaver–Burk plots. All experiments were performed in triplicate to ensure reproducibility 46.

(2) $$\frac{1}{\;V}=\frac{K_{m}}{V_{\max}}\frac{1}{\left[S\right]}+\frac{1}{V_{\max}}$$

All measurements were performed in triplicate. Moreover, BALP kinetic parameters were determined in the presence of different concentrations of TiO_2_ nanoparticles.

### 4.7 Molecular docking studies

Due to the lack of a crystallographic structure for BALP in the Protein Data Bank, a homology model was generated using Modeller 9.10 and validated via RAMPAGE Ramachandran plot analysis (RAMPAGE). The structure of *Escherichia coli* alkaline phosphatase (PDB ID: 1ALK, resolution 2.0 Å served as the template), given its structural homology to mammalian ALP. The TiO_2_ nanoparticle structure was obtained from available chemical databases. Docking was performed using PatchDock (http://bioinfo3d.cs.tau.ac.il/PatchDock/), which employs shape complementarity principles. The results were visualized and analyzed using Visual Molecular Dynamics (VMD) and LigPlot + to identify key interaction residues and binding energies.

## Consent for publication

All authors give their consent for the publication of identifiable details in this journal and article.

## Ethics declaration

Not applicable.

## Funding

We DO NOT have a significant financial interest that would reasonably appear to be affected by the proposed research activities.

## CRediT authorship contribution statement

**Nasim Babaknejad:** Conceptualization, Methodology, Software, Writing – original draft, Writing – review & editing. **Behzad Shareghi:** Methodology, Supervision, Writing – review & editing. **Ali Akbar Saboury:** Data curation, Methodology, Supervision, Writing – original draft.

## Declaration of competing interest

The authors declare that they have no known competing financial interests or personal relationships that could have appeared to influence the work reported in this paper.

## Data Availability

Data will be made available on request.

## References

[1] Gupta S.M., Tripathi M. (2011). A review of TiO_2_ nanoparticles. Chin. Sci. Bull..

[2] Gianfreda L., Scarfi M.R. (1991). Enzyme stabilization: state of the art. Mol. Cell. Biochem..

[3] Kim J., Gratea J.W., Wang P. (2006). Nanostructures for enzyme stabilization. Chem. Eng. Sci..

[4] McComb R.B., Bowers G.N., Posen S. (1979).

[5] Butterworth P. (1983). Alkaline phosphatase. Biochemistry of mammalian alkaline phosphatases. Cell Biochem. Funct..

[6] Coleman J.E. (1992). Structure and mechanism of alkaline phosphatase. Annu. Rev. Biophys. Biomol. Struct..

[7] Bortolato M., Besson F., Roux B. (2002). An infrared study of the thermal and pH stabilities of the GPI-Alkaline phosphatase from bovine intestine. Biochem. Biophys. Res. Commun..

[8] Combes D., Yoovidhya T., Girbal E., Willemot R.M., Monsan P. (1987). Mechanism of enzyme stabilization. Ann. N. Y. Acad. Sci..

[9] Millán J.L. (2006). Alkaline phosphatases: structure, substrate specificity and functional relatedness to other members of a large superfamily of enzymes. Purinergic Signal..

[10] Millán J.L. (2006).

[11] Sekiguchi S., Hashida Y., Yasukawa K., Inouye K. (2011). Effects of amines and aminoalcohols on bovine intestine alkaline phosphatase activity. Enzym. Microb. Technol..

[12] Dumitraşcu L., Stănciuc N., Aprodu I., Ciuciu A.M., Alexe P., Bahrim G.E. (2015). Monitoring the heat-induced structural changes of alkaline phosphatase by molecular modeling, fluorescence spectroscopy, and inactivation kinetics investigations. JFST.

[13] Alizadeh Zeinabad H., Kachooei E., Saboury A.A., Kostova I., Attar F., Vaezzadeh M., Falahati M. (2016). Thermodynamic and conformational changes of protein toward interaction with nanoparticles: a spectroscopic overview. RSC Adv..

[14] Sharma A., Qiang Y., Antony J., Meyer D., Kornacki P., Paszczynski A. (2007). Dramatic increase in stability and longevity of enzymes attached to monodispersive iron nanoparticles. IEEE Trans. Magn..

[15] Wang G., Chen Y., Yan C., Lu Y. (2015). Study on the interaction between gold nanoparticles and papain by spectroscopic methods. J. Lumin..

[16] Robin A., Veni V.A., Thanigaivel M. (2012). Delivery of protein using nanoparticles. Int. J. Pharm. Pharmaceut. Sci..

[17] Xiao Q., Huang S., Su W., Li P., Ma J., Luo F., Liu Y. (2013). Systematic investigations of conformation and thermodynamics of HSA adsorbed to different sizes of CdTe quantum dots. Colloids Surf. B Biointerfaces.

[18] Saeidifar M., Mansouri-Torshizi H., Saboury A.A. (2015). Biophysical study on the interaction between two palladium (II) complexes and human serum albumin by Multispectroscopic methods. J. Lumin..

[19] Šutković J., Jašarević A. (2016). A review on Nanoparticle and Protein interaction in biomedical applications. Period. Eng. Nat. Sci..

[20] Pfeiffer C., Rehbock C., Hühn D., Carrillo-Carrion C., de Aberasturi D.J., Merk V., Parak W.J. (2014). Interaction of colloidal nanoparticles with their local environment: the (ionic) nanoenvironment around nanoparticles is different from bulk and determines the physico-chemical properties of the nanoparticles. J. R. Soc. Interface.

[21] McNamara K., Tofail S.A.M. (2016). Nanoparticles in biomedical applications. Adv. Phys. X.

[22] Wang J., Wu J., Zhang Z., Zhang X., Pan Z., Wang L., Xu L. (2006). Sonocatalytic damage of bovine serum albumin (BSA) in the presence of nanometer anatase titanium dioxide (TiO2). Ultrasound Med. Biol..

[23] Li D., Wang S., Wang J., Zhang X., Liu S. (2013). Synthesis of CdTe/TiO2 nanoparticles and their photocatalytic activity. Mater. Res. Bull..

[24] Vergaro V. (2015). Interaction between human serum albumin and different anatase TiO2 nanoparticles: a nano-bio interface study. Nanomater. Nanotechnol..

[25] Momeni L., Shareghi B., Saboury A.A., Evini M. (2017). Interaction of TiO2 nanoparticle with trypsin analyzed by kinetic and spectroscopic methods. Chem. Mon..

[26] Roseenheck K., Doty P. (1961). The far ultraviolet absorption spectra of polypeptide and protein solutions and their dependence on conformation. Proc. Natl. Acad. Sci. USA.

[27] Schmid F.X. (2001). Encyclopedia of Life Sciences.

[28] Jiang G., Abramavicius D., Bulheller B.M., Hirst J.D., Mukamel S. (2010). Ultraviolet spectroscopy of protein backbone transitions in aqueous solution: combined QM and MM simulations. J. Phys. Chem. B.

[29] Hu X., Yu Z., Liu R. (2013). Spectroscopic investigations on the interactions between isopropanol and trypsin at the molecular level. Spectrochim. Acta Mol. Biomol. Spectrosc..

[30] Hu Y., Li W., Liu Y., Dong J.X., Qu S.S. (2005). Fluorometric investigation of the interaction between methylene blue and human serum albumin. J. Pharmaceut. Biomed. Anal..

[31] Lakowiczv J.R. (2006).

[32] Saeidifar M., Mansouri-Torshizi H., Saboury A.A. (2017). Insights into the binding of two antitumor Pd(II) complexes with human serum albumin. JICS.

[33] Chen F., Liu G., Xu Z., Zeng Z. (2008). Effect of metal ions on the secondary structure and activity of calf intestine phosphatase. BMB Rep..

[34] Zhu J., Chen L., Hu W., Zhang S., Wu D., Liu X. (2017). Molecular spectroscopic insight into the binding of batatasin V isomers to human serum albumin. Spectrosc. Lett..

[35] Zhang R., Liu Y., Huang X., Xu M., Liu R., Zong W. (2018). Interaction of a digestive protease, Candida rugosa lipase, with three surfactants investigated by spectroscopy, molecular docking and enzyme activity assay. Sci. Total Environ..

[36] Ross P.D., Subramanian S. (1981). Thermodynamics of protein association reactions: forces contributing to stability. Biochemistry.

[37] Greenfield N.J. (2006). Using circular dichroism spectra to estimate protein secondary structure. Nat. Protoc..

[38] Cheng F.Q., Wang Y.P., Li Z.P., Dong C. (2006). Fluorescence study on the interaction of human serum albumin with bromsulphalein. pectrochimica acta part Spectrochim Acta A Mol Biomol Spectrosc.

[39] Ghosh G., Gaikwad P.S., Panicker L., Nath B.B., Mukhopadhyaya R. (2016). Unfolding and inactivation of proteins by counterions in protein-nanoparticles interaction. Colloids Surf. B Biointerfaces.

[40] Sethuraman A., Vedantham G., Imoto T., Przybycien T., Belfort G. (2004). Protein unfolding at interfaces: slow dynamics of alpha-helix to beta-sheet transition. Proteins.

[41] Xu Z., Liu X.W., Ma Y.S., Gao H.W. (2010). Interaction of nano-TiO2 with lysozyme: insights into the enzyme toxicity of nanosized particles. Environ. Sci. Pollut. Res. Int..

[42] Kelly S.M., Price N.C. (2000). The use of circular dichroism in the investigation of protein structure and function. Curr. Protein Pept. Sci..

[43] Kelly S.M., Jess T.J., Price N.C. (2005). How to study proteins by circular dichroism. Biochim. Biophys. Acta, Proteins Proteomics.

[44] Kotormán M., Laczkó I., Szabó A., Simon L.M. (2003). Effects of Ca2+ on catalytic activity and conformation of trypsin and alpha-chymotrypsin in aqueous ethanol. Biochem. Biophys. Res. Commun..

[45] Reddy P.M., Umapathia R., Venkatesu P. (2015). A green approach to offset the perturbation action of 1-butyl-3-methylimidazolium iodide on α-chymotrypsin. Phys. Chem. Chem. Phys..

[46] Chaudhuri G., Chatterjee S., Venu-Babu P., Ramasamy K., Thilagaraj W.R. (2013). Kinetic behavior of calf intestinal alkaline phosphatase with pNPP. Indian J. Biochem. Biophys..

