# Supplementary material for: Effects of TiO2 nanoparticles on the kinetics and conformation of bovine alkaline phosphatase
Source: Biochem Biophys Rep. 2026 Jul 18;47:102709. doi: 10.1016/j.bbrep.2026.102709 (PMC13400419; doi:10.1016/j.bbrep.2026.102709)
Supplement: Multimedia component 1 [file mmc1.docx]

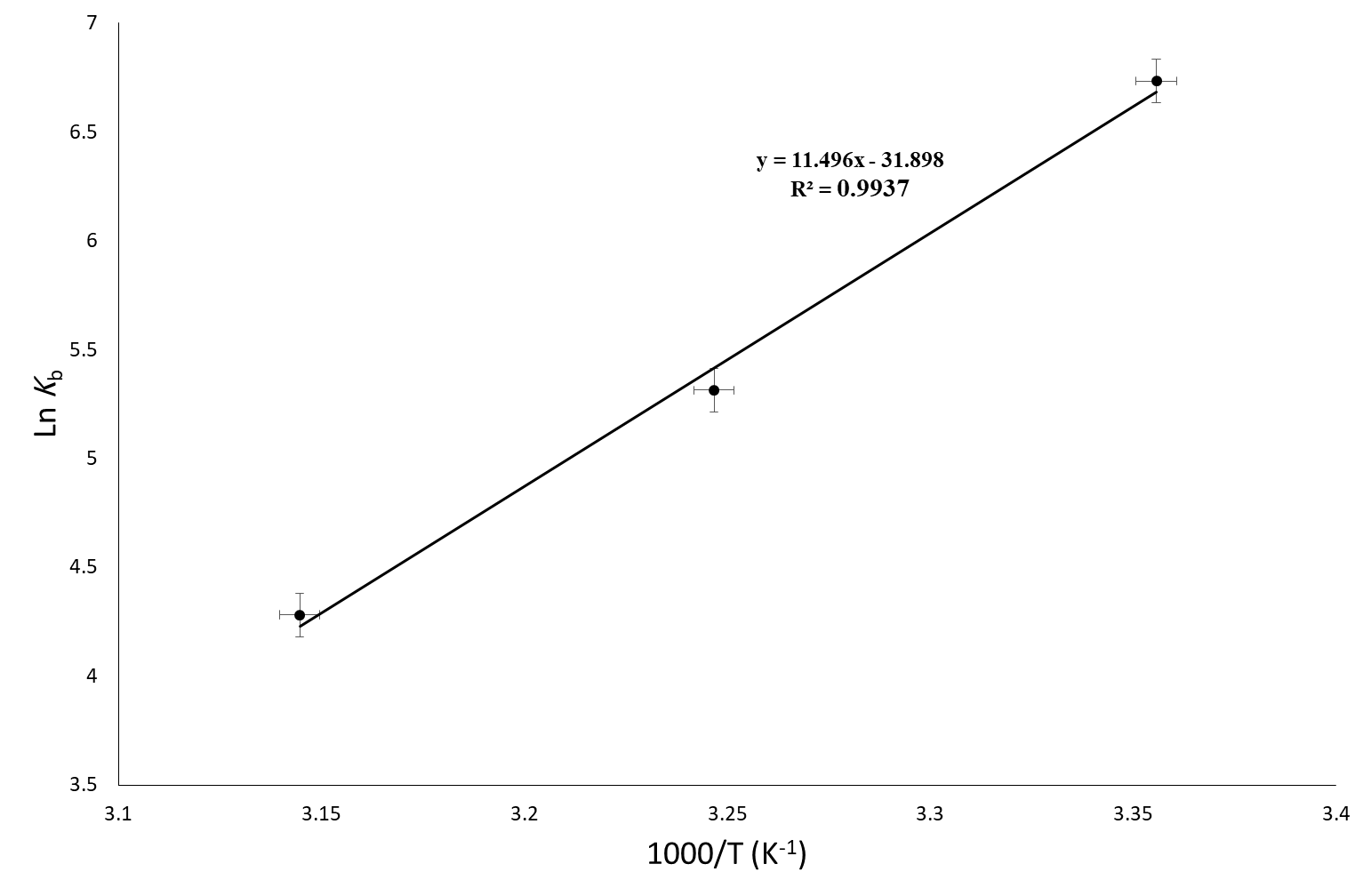


Figure S1. Van’t Hoff plot for the interaction of TiO₂ nanoparticles with bovine alkaline phosphatase


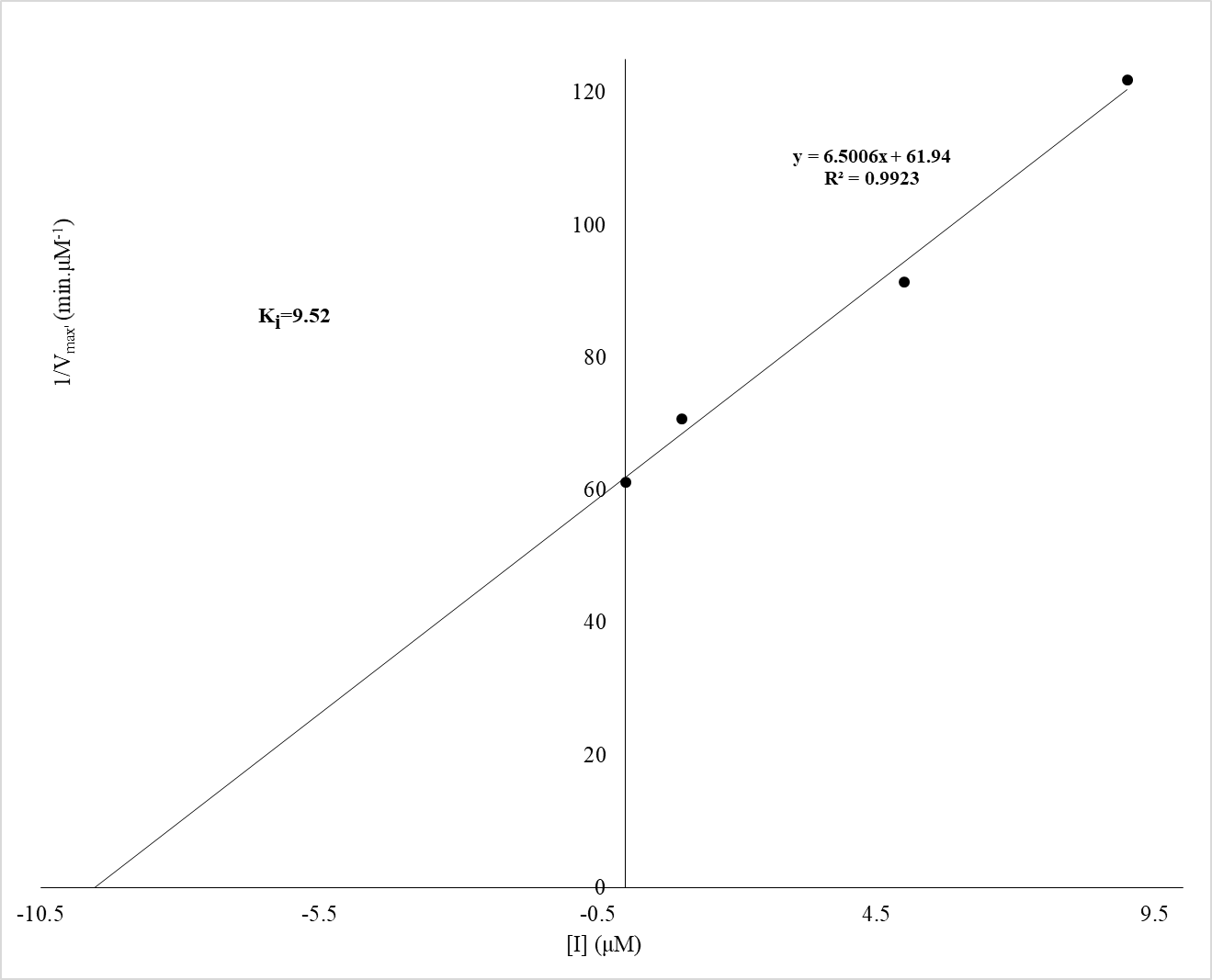


Figure S2. Secondary plot of $\frac{1}{V_{max'}}$versus inhibitor concentration [I].
